# Direct and crossed effects of somatosensory electrical stimulation on motor learning and neuronal plasticity in humans

**DOI:** 10.1007/s00421-015-3248-z

**Published:** 2015-09-03

**Authors:** M. P. Veldman, I. Zijdewind, S. Solnik, N. A. Maffiuletti, K. M. M. Berghuis, M. Javet, J. Négyesi, T. Hortobágyi

**Affiliations:** Center for Human Movement Sciences, University of Groningen, University Medical Center Groningen, Antonius Deusinglaan 1, 9713AV FA23 Groningen, The Netherlands; Department of Neuroscience, University of Groningen, University Medical Center Groningen, Groningen, The Netherlands; Motor Control Laboratory, Department of Kinesiology, Pennsylvania State University, State College, PA USA; University School of Physical Education, Wroclaw, Poland; Neuromuscular Research Laboratory, Schulthess Clinic, Zurich, CH Switzerland; Department of Health Sciences and Technology, ETH Zurich, Zurich, Switzerland; Department of Biomechanics, Kinesiology, and Informatics, University of Physical Education, Budapest, Hungary; Faculty of Health and Life Sciences, Northumbria University, Newcastle-upon-Tyne, UK

**Keywords:** Corticospinal excitability, Interlimb transfer, Motor evoked potential, Primary motor cortex, Transcranial magnetic stimulation

## Abstract

**Purpose:**

Sensory input can modify voluntary motor function. We examined whether somatosensory electrical stimulation (SES) added to motor practice (MP) could augment motor learning, interlimb transfer, and whether physiological changes in neuronal excitability underlie these changes.

**Methods:**

Participants (18–30 years, *n* = 31) received MP, SES, MP + SES, or a control intervention. Visuomotor practice included 300 trials for 25 min with the right-dominant wrist and SES consisted of weak electrical stimulation of the radial and median nerves above the elbow. Single- and double-pulse transcranial magnetic stimulation (TMS) metrics were measured in the intervention and non-intervention extensor carpi radialis.

**Results:**

There was 27 % motor learning and 9 % (both *p* < 0.001) interlimb transfer in all groups but SES added to MP did not augment learning and transfer. Corticospinal excitability increased after MP and SES when measured at rest but it increased after MP and decreased after SES when measured during contraction. No changes occurred in intracortical inhibition and facilitation. MP did not affect the TMS metrics in the transfer hand. In contrast, corticospinal excitability strongly increased after SES with MP + SES showing sharply opposite of these effects.

**Conclusion:**

Motor practice and SES each can produce motor learning and interlimb transfer and are likely to be mediated by different mechanisms. The results provide insight into the physiological mechanisms underlying the effects of MP and SES on motor learning and cortical plasticity and show that these mechanisms are likely to be different for the trained and stimulated motor cortex and the non-trained and non-stimulated motor cortex.

## Introduction

Sensory inputs from the environment provide feedback for the motor system to accurately perform motor tasks and are essential for motor learning (Gentilucci et al. 1997; Rosenkranz and Rothwell 2012). In contrast, reduced sensory function results in decreased manual motor function (Rothwell et al. 1982) and interferes with the recovery of voluntary movements after a stroke (Nudo et al. 2000). These observations led to the idea that enriched compared with normal sensory inputs could augment motor performance. Indeed, several studies reported increases in performance after mild, low-intensity peripheral nerve stimulation, i.e., somatosensory electrical stimulation (SES), but almost exclusively in patients with neurological disorders (Celnik et al. 2007; Conforto et al. 2007; Koesler et al. 2009; Sawaki et al. 2006; Sorinola et al. 2012; Wu et al. 2006).

The mechanisms of how SES improves motor performance and if it could have non-focal crossed effects are not entirely clear. Neuroanatomical, imaging, neuromagnetic, and electrophysiological studies revealed increased activation of the contralateral primary sensory cortex (S1), supplementary motor area, dorsal premotor cortex, posterior parietal cortex, and secondary sensory cortices (S2) bilaterally after SES (Allison et al. 1989, 1991; Forss et al. 1994; Golaszewski et al. 2004; Hari et al. 1984, 1990; Manto et al. 2006; Rosen and Asanuma 1972; Wu et al. 2005). In addition, the excitability of the corticospinal path as evaluated by the amplitude of motor evoked potentials (MEPs) induced by transcranial magnetic stimulation (TMS) increased after bouts of SES in the stimulated (Charlton et al. 2003; Kaelin-Lang et al. 2002; Mang et al. 2011; McKay et al. 2002; Ridding et al. 2000, 2001) and homologous contralateral muscles (Shin and Sohn 2011), confirming that unilateral SES can have non-focal, bilateral effects.

That motor practice and SES administered individually would activate similar structures, raised the possibility that SES could have an additive effect in the SES-stimulated muscles (i.e., direct effects) when combined with motor practice. That is, SES may upregulate the excitability of neurons also accessed by motor practice because of direct connections between SES-activated sensorimotor areas and the primary motor cortex (M1). To strengthen this hypothesis, motor practice, in addition to SES, also increases corticospinal excitability (Jensen et al. 2005; Perez et al. 2004). In addition, it is also possible that due to its bilateral effects on putative sensorimotor areas, SES could have an additive (i.e., crossed effects) effect in the non-stimulated muscles (Veldman et al. 2014). Therefore, the purpose of the present study was to examine the possibility that SES added to motor practice could augment motor learning, interlimb transfer, and neuronal excitability in healthy adults. To address potential mechanisms underlying the direct, crossed, and additive effects of SES, we measured corticospinal excitability, short-interval intracortical inhibition (SICI), intracortical facilitation (ICF), interhemispheric inhibition (IHI), and contralateral facilitation (CLF) in the left and right M1 by means of TMS.

## Materials and methods

### Participants and ethical approval

Thirty-one healthy right-handed volunteers (age 22 ± 3 years, 16 men) agreed to participate in this study. Handedness was determined using the Edinburgh Handedness Inventory (Oldfield 1971). Based on health-related and TMS questionnaires (Rossi et al. 2009), participants had no history of neurological disorders, were not taking drugs that affected functioning of the central nervous system, or had no contraindications for TMS. Every participant included in the study signed a written informed consent. The experiments were conducted according to the declaration of Helsinki and the Medical Ethical Committee of the University Medical Center Groningen approved the experimental protocol and the study was registered at the Dutch trial register (NTR4397).

### Experimental design

**Fig. 1:**
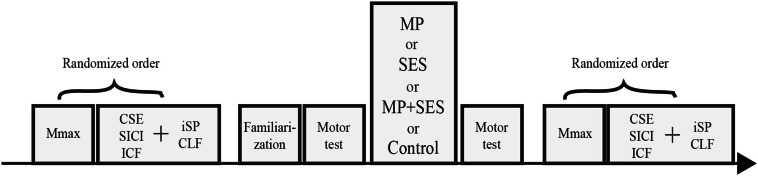
Schematic overview of the experimental design. Baseline measurements including maximal compound action potentials (Mmax), corticospinal excitability (CSE), short-interval intracortical inhibition (SICI), intracortical facilitation (ICF), contralateral facilitation (CLF), and ipsilateral silent period (iSP) were performed before familiarization of the visuomotor task and after completion of one of the three interventions and motor tests

After meeting the inclusion criteria, participants were randomly assigned to one of three intervention groups: motor practice (MP; *n* = 8; 4 men; 23.6 ± 3 years; 1.77 m; 71.8 kg); SES (*n* = 8; 4 men; 21.9 ± 2 years; 1.79 m; 73.2 kg); MP + SES (*n* = 9; 5 men, 20.7 ± 2 years; 1.82 m; 77 kg); or Control (*n* = 6; 3 men; 22.0 ± 2 years; 1.75 m; 70.8 kg) (Fig. 1). Before the start of the intervention, baseline measures were performed by means of TMS and peripheral electrical nerve stimulation. Familiarization with the motor task consisted of three visuomotor trials with each hand before the behavioral testing started. As a control group to control for testing effects, six participants performed familiarization and behavioral measures without any intervention.

### Behavioral testing

Performance in the visuomotor task was the behavioral outcome. Participants sat in a chair without armrests, 90 cm in front of a laptop computer’s monitor (diagonal dimension 40 cm). With the thumbs superior, participants placed the half-supinated right or left hand inside a padded manipulandum. The center of the wrist joint was aligned with the axis of the manipulandum. The device allowed participants only to flex and extend the wrist in the sagittal plane. The resting hand was placed on the table in a half-supinated position, covered with soft material for the comfort of the participants. The feet were on the floor with the knees flexed 90°.

Visuomotor performance was tested using 12 trials before and after each of the three interventions or control period. As in previous studies using the ankle, elbow, and metacarpophalangeal joints, participants followed a preprogrammed template as accurately as possible by flexing and extending their wrist (Cirillo et al. 2011; Jensen et al. 2005; Perez et al. 2004). Custom software-generated templates appeared in the middle of the monitor’s left side in white over a dark blue background in high resolution. The templates progressed from left to right at a speed of 3.3–4.0 cm/s. To make the visuomotor target more challenging to follow, wrist flexion and extension appeared, respectively, as downward and upward deviation on the monitor. In total, there were six different templates that appeared in random order and duration, varying between 4 and 6 s, on the screen but each participant received the same set of templates before and after the intervention.

#### MP intervention

MP consisted of 300, 5-s-long visuomotor intervention trials with the right hand that differed from the test trials. Both intervention and test templates had, on average, seven turns (i.e., changes in direction) and varied randomly in duration between 4 and 6 s. The intervention trials were divided in five blocks of 60 trials, with 2 min of rest between blocks. After every 15 trials, participants were asked to count backwards by seven, starting from a randomly determined two-digit number to keep attention high. To rule out the effects of having electrodes attached to the skin on experimental outcomes, two electrodes (ConMed Cleatrode, AG/AgCl, Ref 1720-003, NY, USA) were placed over the radial and median nerve of the right arm above the elbow but no electrical current was applied.

#### SES intervention

Surface electrodes were placed as described for the MP intervention. A constant-current stimulator (Digitimer Ltd model DS7A, Welwyn Garden City, UK) was programmed to deliver 500 ms trains of electrical stimuli continuously, with one train per second (duty cycle 50 %). Each train consisted of five square wave pulses delivered at 10 Hz (pulse width, 1 ms) (Ridding et al. 2000). At 1 ms pulse width, sensory fibers have a lower threshold than motor fibers whereas at a shorter pulse width, motor fibers have a lower threshold compared to sensory fibers. Therefore, SES as used in the present study activated predominantly cutaneous and proprioceptive fibers (Panizza et al. 1992). Stimulus intensity was set at twice the perceptual threshold (2.8 ± 2.1 mA), determined as the lowest stimulation intensity perceived by the participant. Participants in this group sat in front of a table and looked at the computer monitor while receiving stimulation in five blocks of 5 min (1500 trains and 7500 pulses in total) and performed the backward counting attention task during which SES was paused. The SES parameters selected for the present study were based on clinical studies that reported increases in motor performance and corticospinal excitability after a period of SES (Koesler et al. 2009; Sawaki et al. 2006; Wu et al. 2006).

#### MP + SES intervention

This group received SES concurrently with MP. The details of this combined MP + SES protocol were identical to the details of the individual MP and SES protocols.

### EMG recording

Surface electromyography (EMG) was recorded from the left and the right extensor carpi radialis (ECR) using 37 × 26 × 15 mm, 14 g, wireless, pre-amplified parallel-bar sensors, affixed to the skin with a four-slot adhesive skin interface (Trigno, Delsys Inc, Natick, MA, USA). The EMG signal was recorded with a bandwidth of 20–450 Hz, amplified 909 times, with a channel noise less than 0.75 µV, and a common mode rejection ratio over 80 dB. To minimize noise in the EMG signal, the skin over the muscle belly was shaved, scrubbed with sandpaper, and cleaned with alcohol. EMG activity was sampled at 4 kHz and EMG signals were recorded using data acquisition software (Power 1401 and Signal, Cambridge Electronics Design, Cambridge, UK). The data were stored on a personal computer for off-line analysis.

### Transcranial magnetic brain stimulation

Motor evoked potentials were evoked by a figure of eight-shaped magnetic coil connected to two Magstim 200 magnetic stimulators through a BiStim module (loop diameter, 9 cm; Magstim, Dyfed, UK). MEPs were obtained in all participants except for one participant in SES and all control participants. The coil was placed over the motor area of the right and left hand with the handle pointing backwards at ~45° away from the sagittal plane. The optimal spot, the hotspot to evoke MEPs in the ECR was marked on a cloth cap worn by the participants to ensure consistent repositioning of the coil. Resting motor threshold (rMT) was determined in a sitting position to the nearest 1 % of the maximum stimulator output that evoked MEPs in the ECR of at least 50 μV in five out of ten subsequent stimuli.

Corticospinal excitability, SICI and ICF were measured in one TMS run, delivered in a random order with 10 % variation in 5-s inter-pulse time to reduce anticipation by the participant. To evoke SICI and ICF, a paired-pulse TMS protocol was used as described previously (Kujirai et al. 1993). A subthreshold conditioning stimulus set at 80 % of rMT was delivered 2 ms for SICI and 10 ms for ICF before a suprathreshold test stimulus set at 120 % of the rMT. In all groups, there were ten corticospinal excitability, ten SICI, and ten ICF trials, delivered with at least 5 s between trials at a constant TMS intensity regardless of changes in excitability (Garry and Thomson 2009).

In a separate TMS run, iSP and CLF were measured. First, maximum voluntary contractions in both ECR muscles were determined. With the test stimulus set at 160 % of rMT, five TMS pulses were given with both hands at rest. Thereafter, TMS pulses were given to the M1 while the hand ipsilateral to the TMS stimulus hand was contracted at 20 % of the maximum voluntary force, evoking an iSP in the contracting hand.

### Peripheral electrical nerve stimulation

Maximum compound action potentials (Mmax) were evoked in the left and right ECR by stimulating the radial nerve above the elbow with a single pulse (pulse width 1 ms) by means of the same stimulator used for SES. Stimulation intensity was increased from a subthreshold level to an intensity at which the peak-to-peak amplitude of the *M* wave was no longer increasing. An extra pulse at 120 % of this intensity was given to ensure a plateau was reached. The purpose of this measurement was to normalize MEPs by Mmax, thus enabling the comparison between pre- and post-intervention measures.

### Data analysis

Visuomotor performance was calculated as the mean absolute deviation from the preprogrammed template using a Matlab script (Mathworks, Natick, Massachusetts, USA). To determine if the intervention differently affected the magnitude of learning, the mean absolute error for the 12 pre-intervention trials was compared to the mean absolute error for the 12 post-test trials.

We quantified peak-to-peak amplitude of each MEP recorded in the right and left ECR. MEPs that differed from the mean by more than two standard deviations were excluded for every participant separately. In total, 7 % of all MEPs were excluded. We compared Mmax-normalized MEPs before and after the intervention (corticospinal excitability = MEP/Mmax). Conditioned MEPs were expressed as a percent of test MEP size (SICI = conditioned MEP/test MEP; ICF = conditioned MEP/test MEP). Lower values for SICI and ICF represent more inhibition and less facilitation, respectively.

Onset, offset, and duration of iSP were determined using an adjusted version of the Teager–Kaiser Energy Operator (Solnik et al. 2010), detecting disruption in the ongoing EMG activity. This statistical method uses the signal and noise elements and an upper and lower variation limit in the EMG recording ± (MCD × 2.22). MCD represents the mean consecutive difference of prestimulus EMG points for each individual. The value 2.22 corresponds to 2.5 times the SD and gives a measure of the 98.7 % variation limits of the prestimulus EMG.

Contralateral facilitation was calculated as a ratio between the MEP size during contraction of the hand ipsilateral to the TMS stimulus and MEP size with this hand at rest (CLF = MEP 20 %MVC/MEP rest). Background EMG activity was determined after rectifying the EMG signal. The relation between associated activity in the ‘resting’ hand and facilitation of the MEP size during contraction of the hand ipsilateral to the TMS stimulus was determined using correlation analysis.

### Statistical analysis

All data were checked for normal distribution using the Shapiro–Wilk test. Log transformation was used for variables that revealed not normal. The analyses were done on the transformed data using SPSS (version 22.0) but all variables are reported in their original, non-transformed, form as mean ± standard deviation.

Visuomotor performance, MEPs, SICI, ICF, IHI, and CLF pre- and post-intervention were compared by a three (Group) by two (Time) repeated measures analysis of variance (ANOVA) on Time for each side (Left, Right) separately. In case of a significant *F* value for the Group by Time interaction effect, Tukey’s post hoc calculations for repeated measures ANOVA were performed to identify means that differed at *p* < 0.05. A Greenhouse–Geisser correction was used when the assumption of sphericity was violated. Pearson correlation analysis was used to identify significant relationships between behavioral and neurophysiological variables at *p* < 0.05.

## Results

### Behavioral data

**Fig. 2:**
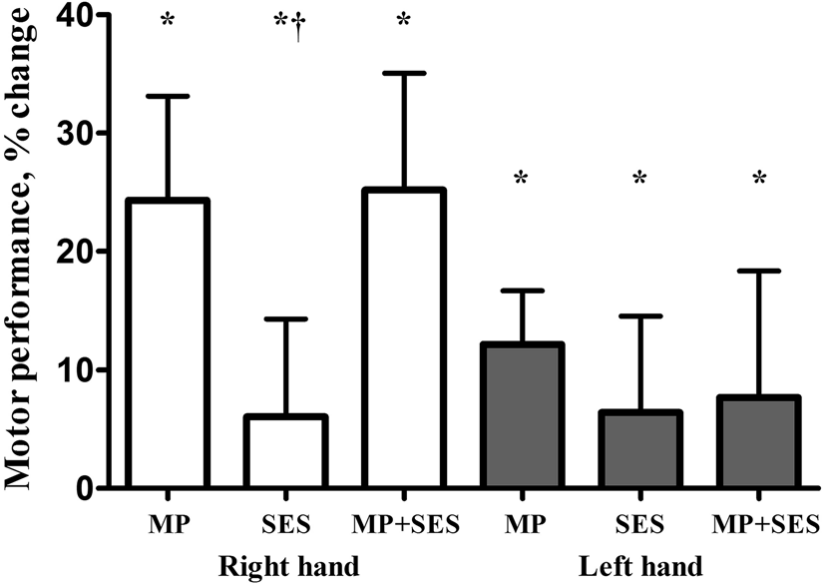
Increases in motor performance after motor practice (MP), somatosensory electrical stimulation (SES), and MP + SES in the intervention (*open bars*) and non-intervention (*filled bars*). Motor performance was computed as a reduction in template-matching errors. Performance improved more after MP and MP + SES in the right hand compared to SES. *Asterisk*, significant Time main effect (*p* < 0.05, *open and filled bars*, respectively, pooled, not graphed); *dagger*, significant Group by Time interaction (*p* < 0.05). *Vertical bars* denote +1SD

Figure 2 shows the Group by Time interaction (*F*_2,22_ = 9.7, *p* = 0.001) in motor learning. All three groups improved motor performance but the decrease in error was greater (*p* < 0.05) in MP (7.7°) and MP + SES (6.7°) compared with SES (2.9°). Figure 2 also shows that the magnitude of transfer of the learned skill after a right-hand visuomotor intervention to the non-intervention left hand was similar: 4.4° (MP), 3.0° (SES), and 3.2° (MP + SES) (Time main effect, *F*_2,22_ = 110.1, *p* < 0.001). The control group showed 2.1° (10 %) and 2.5° (10 %) less error in the intervention and non-intervention hand, respectively. In the remainder of the paper, we report the learning and transfer data adjusted for the effects of familiarization by subtracting the familiarization effects from the effects produced by the interventions. Tables 1 and 2 summarize the absolute and percent changes in motor learning.

**Table 1:** Intervention effects on motor learning in the intervention right and non-intervention left hand

	Pre	Post
	Mean (±SD)	Mean (±SD)
Right		
MP	19.6 (2.2)	11.8 (1.5)
SES	17.8 (1.9)	14.9 (1.8)
MP + SES	18.2 (3.9)	11.5 (1.4)
Control	22.5 (1.8)	20.4 (4.1)
Left		
MP	19.8 (1.8)	15.4 (1.3)
SES	17.7 (2.7)	14.7 (1.4)
MP + SES	17.0 (2.5)	13.8 (1.4)
Control	25.0 (0.6)	22.4 (1.9)

Values are in degrees, expressing the mean absolute error from the target

*MP* motor practice, *SES* somatosensory electrical stimulation, *MP* *+* *SES* motor practice combined with somatosensory electrical stimulation

**Table 2:** Summary of percent changes in motor learning and TMS metrics in the right intervention and left non-intervention M1

	MP	SES	MP + SES
Right hand or left M1			
Motor learning^†^	29.3*	6.1*	25.2*
CSE^†^	43.6*	63.4*	18.9*
SICI	16.4	21.3	1.6
ICF	21.4	14.7	1.5
IHI^†^	14.2*	−7.9*	−1.1
CLF^†^	34.1*	−14.1*	1.1
Left hand or right M1			
Motor learning	12.2*	6.4*	7.7*
CSE^†^	1.3	54.2*	−13.7
SICI^†^	6.1	−21.8*	41.4*
ICF	21	−3.4	−2.2
IHI	−9.5	−6.5	−1.8
CLF	3	−0.2	−14.8

Values are mean percent changes based on individually computed changes

*MP* motor practice, *SES* somatosensory electrical stimulation, *CSE* corticospinal excitability, *SICI* short-interval intracortical inhibition (positive change denote decreases in inhibition), *ICF* intracortical facilitation, *IHI* interhemispheric inhibition (positive change denote reductions in inhibition), *CLF* contralateral facilitation

* *p* < 0.05 based on Tukey’s post hoc test; ^†^ group by Time interaction

### Corticospinal excitability data

**Fig. 3:**
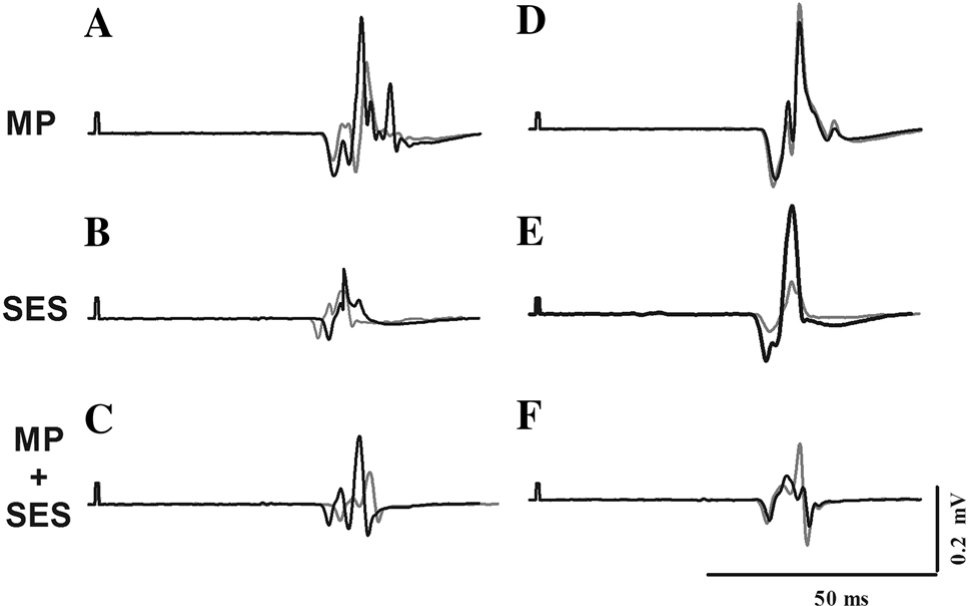
Raw data of changes in corticospinal excitability after motor practice (MP) and somatosensory electrical stimulation (SES). Representative 10-trial-averaged motor evoked potentials (MEPs) measured in the extensor carpi radialis (ECR) representing changes in corticospinal excitability before (*gray lines*) and after (*black lines*) the three interventions in the intervention left M1 (**a**–**c**) and non-intervention right M1 (**d**–**f**)

**Fig. 4:**
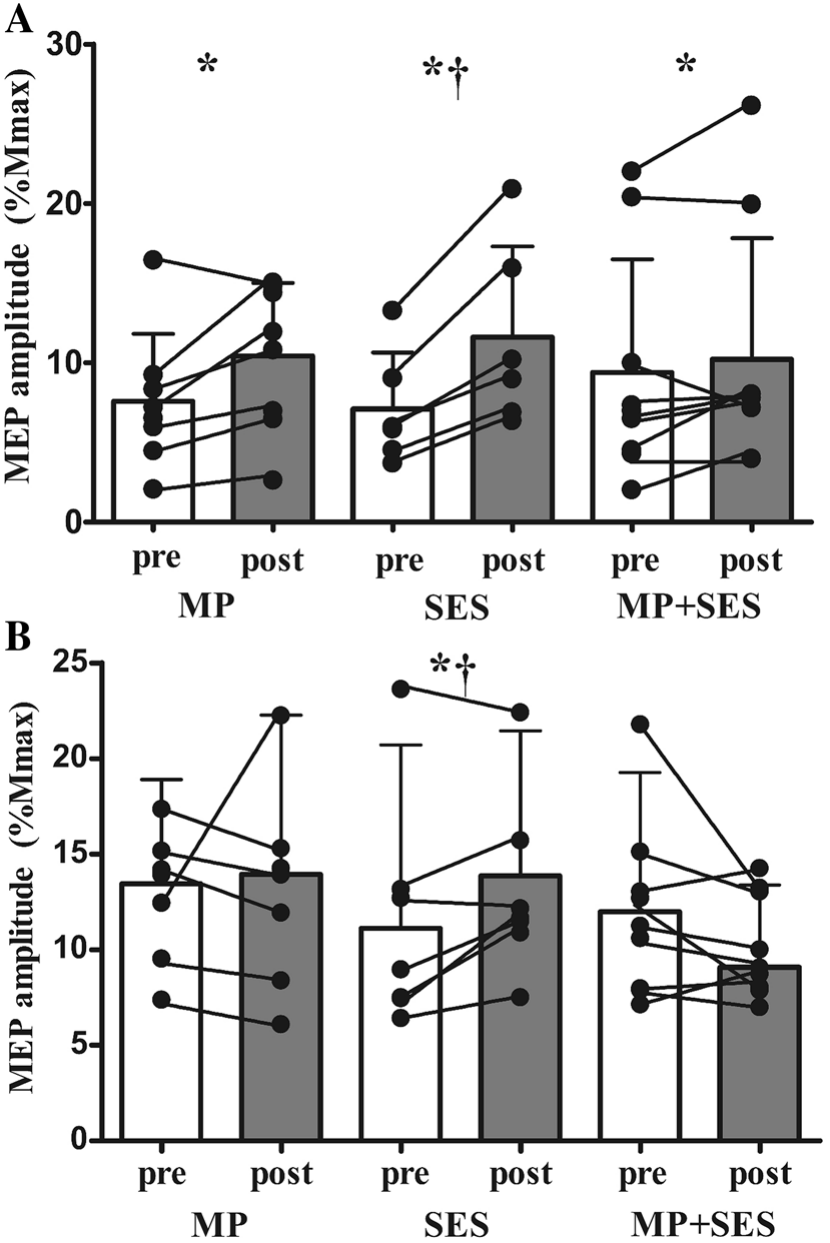
Corticospinal excitability increases in all groups in the intervention M1 and after somatosensory electrical stimulation (SES) in the non-intervention M1. Corticospinal excitability before (*open bars*) and after (*filled bars*) the three interventions in the intervention left M1 (*Panel A*) and non-intervention right M1 (*Panel B*). Corticospinal excitability increased more after SES compared to MP and MP + SES in both M1s. *Interconnected dots* represent individual changes and *vertical bars* denote +1SD. *Asterisk* significant Time main effect (*p* < 0.05); *dagger* significant Group by Time interaction (*p* < 0.05)

Figure 3 shows MEPs in the left M1 (Fig. 3a–c) and right M1 (Fig. 3d–f) in a representative participant in each group. Figure 4a shows the Group by Time interaction in the Mmax-normalized MEPs: the 44 and 63 % increase in MP and SES, respectively, were greater than the 19 % increase in MP + SES (Group by Time interaction, *F*_2,19_ = 3.9, *p* = 0.039). Figure 4b shows that changes in MEPs in the non-intervention right M1 were higher in SES (54 %) compared with MP (1 %) and MP + SES (−14 %) (Group by Time interaction, F_2,20_ = 4.6, *p* = 0.023). None of the interventions modulated the magnitude of the Mmax in the intervention right hand (MP 3.8–4.0 mV; SES 2.8–3.0 mV; MP + SES 4.7–4.6 mV; all *p* > 0.05) and the non-intervention left hand (MP 4.3–4.2 mV; SES 3.1–3.2 mV; MP + SES 3.6–3.7 mV; all *p* > 0.05). Tables 2 and 3 summarize the relative and absolute changes in corticospinal excitability.

**Table 3:** Effects of three interventions on corticospinal and intracortical excitability

	Pre	Post
	Mean (±SD)	Mean (±SD)
*Right M1*		
CSE		
MP	7.6 (4.2)	10.4 (4.6)
SES	7.1 (3.6)	11.6 (5.7)
MP + SES	9.4 (7.1)	10.2 (7.6)
SICI		
MP	28.6 (20.2)	70.9 (43.1)
SES	49.6 (14.2)	60.1 (21.3)
MP + SES	58.0 (33.5)	54.5 (31.7)
ICF		
MP	133.2 (50.6)	142.8 (31.6)
SES	165.1 (78.9)	164.6 (43.2)
MP + SES	145.1 (61.3)	136.2 (48.6)
*Left M1*		
CSE		
MP	13.4 (5.5)	13.9 (8.3)
SES	11.1 (9.6)	13.9 (7.6)
MP + SES	12.0 (7.3)	9.1 (4.3)
SICI		
MP	51.2 (25.8)	54.6 (33.0)
SES	58.5 (15.1)	45.5 (18.3)
MP + SES	58.5 (26.0)	73.8 (31.2)
ICF		
MP	133.5 (39.6)	158.7 (44.7)
SES	135.2 (19.5)	128.7 (17.1)
MP + SES	175.8 (99.0)	157.6 (69.1)

**Fig. 5:**
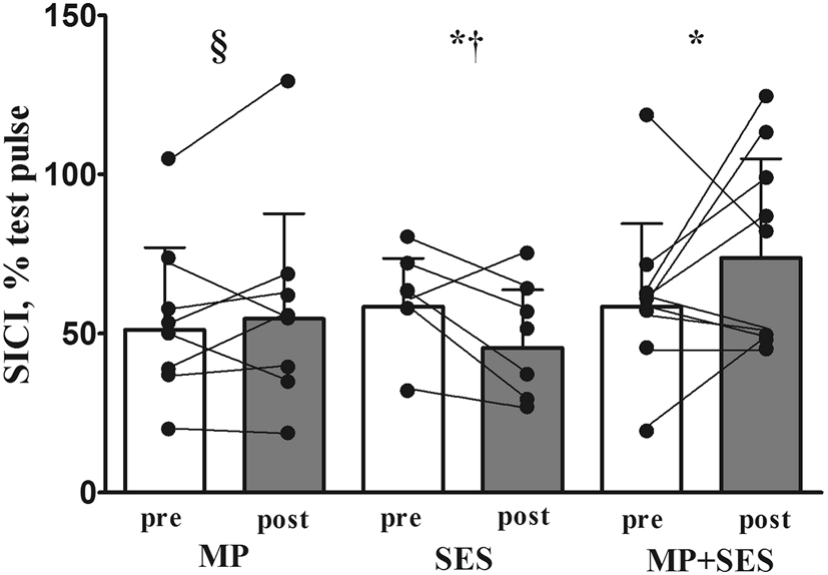
Group and individual changes in short-interval intracortical inhibition (SICI) after motor practice (MP), somatosensory electrical stimulation (SES), and MP + SES in the non-intervention right M1. Conditioned motor evoked potentials (MEP) before (*open bars*) and after (*filled bars*) the three interventions. Lower values for SICI represent higher intracortical inhibition. Group data show that SICI increased after SES and decreased after MP + SES in the non-intervention M1 while MP did not modify SICI. *Interconnected dots* represent individual changes and *vertical bars* denote +1SD. *Asterisk* significant Time main effect (*p* < 0.05); *dagger* and *section sign*, significant Group by Time interaction (*p* < 0.05)

Figure 4a, b illustrates the significant interaction effects for corticospinal excitability in the left and right M1, respectively. Figure 5 denotes a significant interaction effect for SICI in the right M1

*CSE* corticospinal excitability (% maximal compound action potential), *SICI* short-interval intracortical inhibition (% test pulse size), *ICF* intracortical facilitation (% test pulse size)

### Intracortical excitability data

The interventions did not modify SICI (Group by Time interaction, F_2,21_ = 0.6, *p* = 0.565) in the intervention left M1. Figure 5 shows that the three interventions modified SICI differently in the non-intervention right M1 (Group by Time interaction, *F*_2,21_ = 4.2, *p* = 0.028): the 41 % decrease in inhibition after MP + SES (41 %) was greater (*p* < 0.05) than the 6 and −22 % in MP and SES, respectively, with these two latter values also different from one another (*p* < 0.05). There were no changes in ICF in either hemisphere. Tables 2 and 3 summarize the relative and absolute changes in intracortical excitability.

### Interhemispheric excitability data

**Fig. 6:**
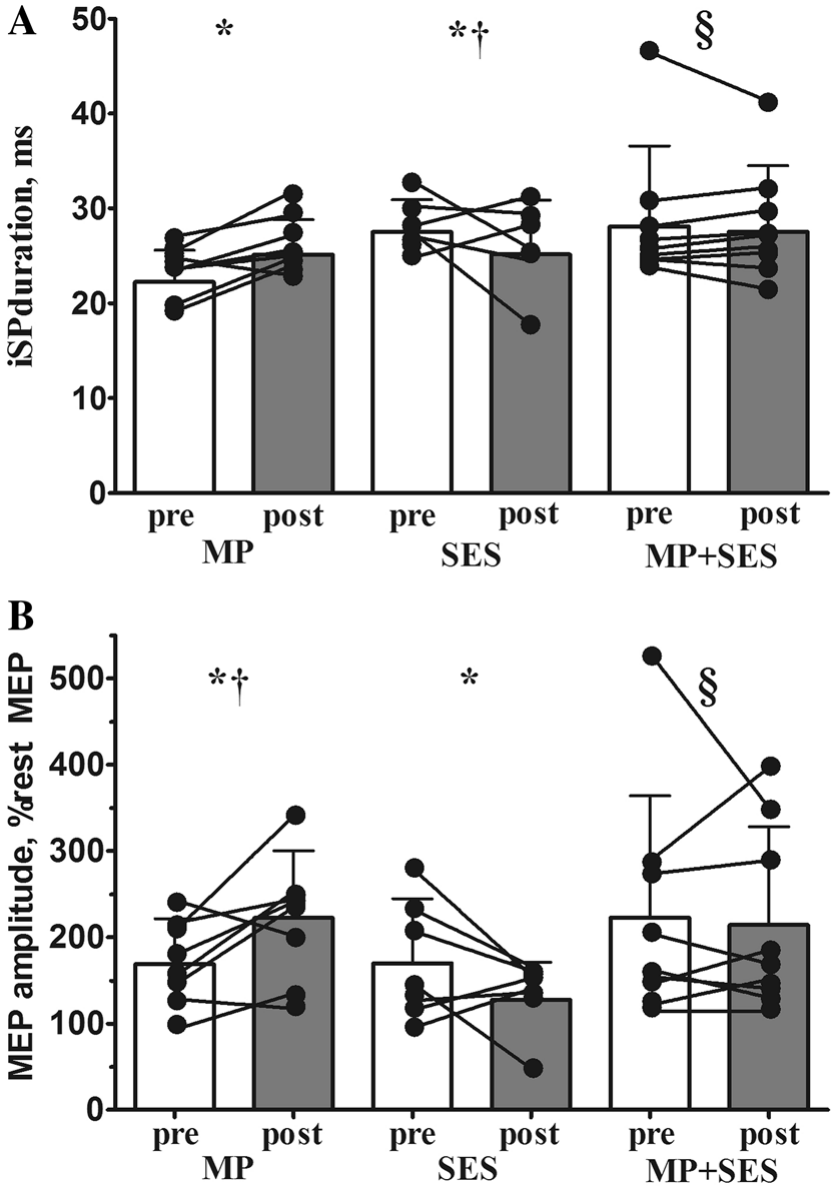
Group and individual changes in interhemispheric inhibition (IHI) and facilitation (CLF) after motor practice (MP) and somatosensory electrical stimulation (SES). IHI and CLF from the left M1 to the right M1 graphed before (*open bars*) and after (*filled bars*) the three interventions. IHI decreased after SES but increased after MP, resulting in a cancelation effect after MP + SES (*Panel A*). Opposite effects were found for CLF (*Panel B*). *Interconnected dots* represent individual changes and *vertical bars* denote +1SD. *Asterisk* significant Time main effect (*p* < 0.05); *dagger* and *section sign* significant Group by Time interaction (*p* < 0.05)

Figure 6a shows that the 8 % longer iSP duration, reflecting higher IHI after MP was greater than the −1 % change after MP + SES and the −14 % shortening of iSP after SES (Group by Time interaction, *F*_2,20_ = 3.7, *p* = 0.044). In contrast, iSP duration remained unchanged in the non-intervention right M1 (*F*_2,18_ = 0.2, *p* = 0.789).

During contraction of the left ECR, there is some associated activity in the ‘resting’ right ECR. We quantified if the interventions modified the magnitude of this associated activity and also the facilitation of MEPs produced by TMS. Figure 6b shows that the three interventions modified this facilitation differently (Group by Time interaction, *F*_2,21_ = 3.6, *p* = 0.044): facilitation was similar after MP (14 %) and MP + SES (1 %) but greater than after SES (−8 %). Also, the unchanged facilitation after MP + SES (i.e., 1 %) was different from the 8 % decrease after SES. The interventions did not modify the MEP facilitation in the left ECR during right ECR contraction (*F*_2,21_ = 1.0, *p* = 0.379). Tables 2 and 4 summarize percent and absolute changes in interhemispheric data.

**Table 4:** Effects of three interventions on interhemispheric excitability

	Pre	Post
	Mean (±SD)	Mean (±SD)
*Left M1*		
iSP		
MP	22.2 (3.4)	25.1 (3.7)
SES	27.5 (3.4)	25.2 (5.7)
MP + SES	28.1 (8.5)	27.6 (7.0)
CLF		
MP	169.0 (52.5)	222.5 (77.5)
SES	170.1 (74.3)	127.8 (43.5)
MP + SES	222.8 (141.4)	214.1 (113.9)
*Right M1*		
iSP		
MP	25.7 (4.8)	22.4 (4.0)
SES	24.7 (1.8)	24.1 (6.2)
MP + SES	27.2 (7.4)	24.9 (5.3)
CLF		
MP	198.5 (81.1)	204.6 (113.5)
SES	177.5 (78.8)	164.6 (41.3)
MP + SES	188.8 (68.7)	153.4 (34.8)

Figure 6a, b illustrates the significant interaction effects for ipsilateral silent period and contralateral facilitation in the left M1, respectively

*iSP* ipsilateral silent period (ms), *CLF* contralateral facilitation (% MEP during contralateral hand contracting and MEP during contralateral hand resting)

### Correlation analyses

Increases in visuomotor performance in the right and left hand did not correlate (*r* = 0.32, *p* = 0.119). Changes in right-hand visuomotor performance did not correlate with increases in corticospinal excitability measured in the left M1 (*r* = −0.17, *p* = 0.210), nor were the changes in left-hand visuomotor performance associated with changes in corticospinal excitability measured in the right M1 (*r* = −0.19, *p* = 0.189). However, increased facilitation of right ECR MEPs during left-hand contraction (CLF) weakly correlated with increased visuomotor performance in the right hand (*r* = 0.45, *p* = 0.013). Changes in iSP did not correlate with increases in visuomotor performance. There was no correlation between the changes in corticospinal excitability in the left M1 and right M1 (*r* = 0.11, *p* = 0.310), but the increase in corticospinal excitability in the right M1 weakly correlated with the decrease in SICI in the right M1 (*r* = −0.37, *p* = 0.039). Also, there was a positive correlation between changes in iSP duration in the left ECR and facilitation of the MEPs in the left ECR during right ECR contractions (CLF; *r* = 0.49, *p* = 0.007). Changes in bEMG in the right ECR did not correlate with changes in facilitation of MEPs in the right ECR during left-hand contractions (CLF; *r* = 0.26, *p* = 0.216).

## Discussion

We found that all three interventions produced motor learning and interlimb transfer but SES added to MP did not further increase learning and transfer (Table 2). Corticospinal excitability strongly increased after MP and SES when measured at rest but it increased after MP and decreased after SES when measured during contraction. No changes occurred in SICI and ICF in the intervention M1. MP did not affect any of the TMS metrics in the non-intervention transfer M1. In contrast, corticospinal excitability strongly increased and SICI strongly decreased in the non-intervention M1 after SES, while MP + SES showed the opposite of these effects. In the non-intervention M1, the increase in corticospinal excitability correlated with decreases in intracortical inhibition. MP and SES affected interhemispheric excitability in the opposite direction. In total, the present study showed that MP and SES each can produce motor learning and interlimb transfer but these effects are non-additive and are likely mediated by different mechanisms.

### Effects of motor practice on motor performance

#### Practice hand

Participants naïve to the task showed 27 % learning in the intervention hand after 25 min of visuomotor practice (Fig. 2). The 27 % learning is comparable with the improvements in the ankle (23 %) (Perez et al. 2004) and metacarpophalangeal joint of the index finger (23 %) (Cirillo et al. 2011) after a similar paradigm and practice duration. The magnitude of learning in the practiced hand is roughly within the range of changes produced by other learning paradigms using a force-control tracking task (36 %) (Floyer-Lea and Matthews 2005). The common element in these single-session learning regimes is that they all represent the rapid, initial phase of motor learning (Dayan and Cohen 2011).

#### Interlimb transfer

Remarkably, practice with voluntary contractions as well as SES, which lacks a voluntary element, both produced interlimb transfer that was statistically similar [Fig. 2, for a review see (Ruddy and Carson 2013)]. The average 9 % net interlimb transfer in the present study was comparable with the 13 % produced by a ball rotation task requiring complex finger movements (Nojima et al. 2012) but both were much smaller than the 62 % produced by 300 ballistic finger movements also completed in one session (Lee et al. 2010). Theories of interlimb transfer imply that the magnitude of transfer is proportional to the magnitude of learning (Ruddy and Carson 2013), a conjecture supported by the *r* = 0.71 (*p* < 0.001) correlation between increases in force of the trained and untrained hand in chronic cross-education studies using low-skill, high-force unilateral muscle contractions [(Zhou 2000), personal communication]. However, this association tends to be lower after rapid tapping movements [*r* = 0.44, *p* = 0.04; (Lee et al. 2010)] or can be absent when the skill is complex as were the case after a ball rotation task (*r* = −0.07, *p* = 0.86; (Nojima et al. 2012), personal communication). The low or altogether absent associations may become stronger after additional practice that consolidates the motor skill into memory (Muellbacher et al. 2002). Alternatively, participants may rely on procedural elements when they perform the task with the non-practice hand, resulting in ‘transfer’. We did notice that learning in the intervention (*r* = 0.53, *p* = 0.006) and in the transfer hand (*r* = 0.59, *p* = 0.002) was associated with the skill level at baseline, suggesting that, if used in a clinical setting, patients with low motor function would benefit most from this type of motor practice. Collectively, the behavioral data are in line with existing evidence suggesting that visuomotor skill practice is an effective and reliable model of motor learning, a model that is now extended to the wrist joint.

### Effects of SES on motor performance

#### Direct and crossed effects of SES

We found some evidence that SES on its own can increase healthy adults’ skill performance by 6 % (Fig. 2; Table 1; *p* = 0.002). Our results are in line with the broad concept that sensory inputs are powerful modulators of motor performance when administered in the form of SES (Sorinola et al. 2012), mirror visual feedback (Nojima et al. 2012), auditory cueing (Brown and Palmer 2013), and muscle warming (Immink et al. 2012). In addition to the direct effects, SES also produced non-focal, crossed effects because the non-intervention hand’s skill performance also improved (6 %, *p* = 0.001). Neuroanatomical, electrophysiological, and imaging data revealed that unilateral electrical stimulation, including SES, can activate the contralateral S1 and S2 bilaterally (Allison et al. 1989; Blickenstorfer et al. 2009; Deuchert et al. 2002; Forss et al. 1994; Golaszewski et al. 2004; Iftime-Nielsen et al. 2012). Direct connections between Brodmann areas 1 and 2 of S1 and M1 (Donoghue and Sanes 1994; Friedman and Jones 1981; Jones 1983; Kaneko et al. 1994), and S2 and M1 (Jones 1983) provide a neuroanatomical basis for the observed effects. The mechanism of how monotonic, non-patterned SES pulses improve complex skills is unclear. Electrical stimulation, however, can facilitate motor learning and skill retention by entraining sensorimotor rhythms (Soekadar et al. 2014) and by having selective effects on oscillatory frequencies underlying motor learning (Joundi et al. 2012). Taken together, the current data provide for the first time evidence that weak electrical nerve stimulation in the form of SES can produce small but statistically and functionally meaningful interlimb transfer in healthy adults.

#### Direct and crossed effects of MP + SES

We also tested if SES combined with MP had an additive effect on skill learning compared with MP or SES. Although MP alone and SES alone increased motor learning by 29 and 6 %, respectively, SES combined with MP did not further increase learning (25 %; Table 2). We expected to find an additive effect because SES activated S1–M1 projections in animal and human brains (Allison et al. 1989, 1991; Jones et al. 1978; Wu et al. 2005) and caused M1 reorganization in rats (Farkas et al. 1999) and healthy humans (Golaszewski et al. 2004; Wu et al. 2005). In addition, combining MP with SES is an effective method to alter sensory states in spinal cord injury patients suffering from sensory deficits (Beekhuizen and Field-Fote 2005, 2008). Notwithstanding these data, we found no additive effect, suggesting that MP might have saturated the circuits SES also accessed, the overlap between the circuits activated by MP and SES was functionally inefficient, or the effects produced by MP and SES interfered with each other. A lack of an additive effect was perhaps also due to the effect of SES alone being small (6 %) in proportion to the 29 % learning produced by MP so that SES could not manifest itself when SES was added to motor practice. An additive effect may still be possible after future studies determine the SES parameters that produce the greatest learning effects.

We also examined the effects of SES combined with MP on interlimb transfer. Our expectation for SES augmenting interlimb transfer was based partly on neuroanatomical paths implicated in such a transfer and on data showing that afferent inputs, in the form of mirror-viewing the hand performing while motor practice, however different from SES, produced 13 % greater skill transfer to the resting hand (Nojima et al. 2012). Despite the plausibility of this hypothesis, we found no evidence for SES augmenting the transfer of a visuomotor skill. Perhaps the level of activation of somatosensory areas ipsilateral to the stimulation by SES was less than reported in previous studies [for a review see (Veldman et al. 2014)], making SES ineffective. It is also possible that MP + SES did not cause additional learning as a result of an interference effect, reflected by opposite adaptations in TMS metrics discussed in following sections. Although the methodological elements in the current study were based on previous studies (Veldman et al. 2014), we demonstrated no additive effect of SES on motor skill learning in the practiced and the non-practiced hand in healthy young adults.

### MP and SES modify corticospinal excitability

#### Direct effects of MP

In agreement with the hypothesis and the prevailing literature, 300 voluntary movements forming 25 min of visuomotor practice increased corticospinal excitability by ~40 % measured at rest [Fig. 4a (Cirillo et al. 2011; Jensen et al. 2005; Perez et al. 2004)]. Increases in MEP size after short-term motor practice are thus common and presumably reflect use-dependent plasticity (Muellbacher et al. 2001; Ziemann et al. 2001) through long-term potentiation-like mechanisms in motor cortical circuits (Butefisch et al. 2000; Classen et al. 1998; Muellbacher et al. 2002). Although it has been reported that M1 is involved in motor learning and early consolidation of motor memories [e.g., (Muellbacher et al. 2002)], increases in corticospinal excitability in the intervention left M1 did not correlate with the behavioral changes in the intervention right hand (*r* = −0.17, *p* = 0.210, *n* = 24), as was also the case in a previous study (Cirillo et al. 2011). A lack of correlation between corticospinal excitability and behavioral changes complements the findings of an earlier study in which 5 Hz repetitive TMS over the M1 reduced corticospinal excitability but did not interfere with motor learning (Shemmell et al. 2007). We speculate that two factors complicate the interpretation of the present and past results and underlie the lack of correlation. One factor is that changes in corticospinal excitability measured at rest may reflect the altered state of circuits that are different from the ones that become active during the learning task. Measurements of corticospinal excitability during the task or muscle contraction may be a more relevant outcome than corticospinal excitability measured at rest. Second, there is a temporal asynchrony between the changes in corticospinal excitability so that the peak changes in each variable occur at different times. Finally, it is also possible that the neurophysiological measures as performed with TMS do not directly reflect changes in excitability essential for motor learning in this context. Despite such caveats, the results of the present and past studies seem all confirm the putative role of M1 in motor learning.

#### Crossed effects of MP

In contrast to the changes seen in the left-intervention M1, corticospinal excitability remained unchanged in the non-intervention right M1 after MP despite evidence for 10 % learning (Fig. 4b) and previous studies reporting increased corticospinal excitability in the non-intervention M1 after ballistic motor practice (Carroll et al. 2008; Lee et al. 2010). Increases in corticospinal excitability may be task-dependent because MEP amplitude in the non-intervention M1 also did not change after a sequence learning task (Perez et al. 2007). Repetitive recruitment of the same corticospinal paths in ballistic motor practice in contrast with tasks involving multiple muscles may explain the differential effects on corticospinal excitability after ballistic motor practice (Baraduc et al. 2004). Corticospinal excitability may not be an optimal neurophysiological marker for motor learning because MP produced transfer without changes in corticospinal excitability, suggesting that structures and/or cortical circuits other than corticospinal paths originating from the M1 ipsilateral to the practicing hand may play a more prominent role in interlimb transfer.

#### Direct and crossed effects of SES

Unlike MP, SES strongly increased corticospinal excitability in both M1 s (Fig. 4a, b; Table 3). Increases in corticospinal excitability after SES have been shown in a range of muscles and body parts (Hamdy et al. 1998; Ridding et al. 2000; Stefan et al. 2000). The magnitude of change in the stimulated hand in the present study was 63.4 % (Table 3), fitting within the range of the 50–96 % increases reported previously (Veldman et al. 2014). Our data provide a hint that the SES adaptations may be non-linear with respect to the duration of the stimulation because we observed a 63 % increase after only 25 min of SES in the ECR, a change crudely similar to 77 % reported after 120 min of SES in the first dorsal interosseous muscle (Charlton et al. 2003; Kaelin-Lang et al. 2002; Ridding et al. 2000; Ridding and Taylor 2001) and abductor digiti minimi muscle (Kaelin-Lang et al. 2002). A plateau of 50 % increase in corticospinal excitability reached after 45 min of SES fits in this non-linear dose–response relationship (McKay et al. 2002). In the non-intervention right M1, corticospinal excitability increased 54.2 %, considerably more than the 9.4 % increase after a paired associative stimulation paradigm delivered at 0.25 Hz for 15 min (Shin and Sohn 2011). Taken together, the present study replicated previous findings showing that SES can increase corticospinal excitability in the SES-stimulated M1 in humans and provide new evidence that SES to a peripheral nerve only increases corticospinal excitability non-focally in the non-intervention M1 considerably.

#### Direct and crossed effects of MP + SES

Against expectations, SES combined with MP did not have an additive effect on corticospinal excitability in the intervention M1. Instead, SES seemed to interfere with MP because MP (43.6 %) and SES (63.4 %) increased corticospinal excitability substantially more than SES combined with MP (18.9 %; Table 3). SES added to chronic massed motor practice in spinal cord injury patients also did not additionally increase corticospinal excitability (Beekhuizen and Field-Fote 2005). Possibly, the combined input is too high for motor cortical neurons to process MP and SES concurrently. MP saturates the corticospinal circuits and leaves no room for SES to additionally increase corticospinal excitability. In the non-stimulated right M1, SES also had no additive effect on corticospinal excitability rather corticospinal excitability actually decreased by 13.7 %. Still, to exploit any potential effects of SES on motor learning, future studies could explore if SES applied as sensory priming before MP could potentiate learning. In sum, combining MP with SES did not have an additive effect on corticospinal excitability in either M1 in healthy young participants probably due to a saturation effect.

### Intracortical excitability

#### Direct effects on SICI

Motor learning (Cirillo et al. 2011; Gallasch et al. 2009; Garry et al. 2004; Perez et al. 2004; Smyth et al. 2010) and SES (Kaelin-Lang et al. 2002) tend to decrease SICI in healthy young adults and patients (range 7–80 %). Reductions in GABA_A_-mediated SICI involve long-term potentiation-like processes (Butefisch et al. 2000) in inhibitory horizontal connections (Hess and Donoghue 1996). Visuomotor training in the first dorsal interosseous and ankle muscles reduced SICI 38 % (Cirillo et al. 2011) and 50 % (Perez et al. 2004), respectively. In the present study, however, we observed a non-significant 13.1 % reduction in SICI in the left-intervention M1 after MP, SES, and MP + SES (Time main effect, *F* = 0.133, *p* = 0.719) and no relationship between the changes in SICI and motor learning (*r* = −0.22, *p* = 0.297) in agreement with previous findings (Cirillo et al. 2011; Garry et al. 2004). There was large variation in the SICI responses among the participants: 12, 3, and 9 of the 24 participants, respectively, showed decreases, no change, or increases. We were also unable to discern subgroups of responders and non-responders to MP or SES in terms of SICI (Murase et al. 2015). Because involvement of long-term potentiation-like processes has been suggested to modify M1 metrics in response to both visuomotor training and SES, perhaps these interventions act on similar brain areas (e.g., premotor cortex) and one would expect a positive interaction between the two types of interventions with respect to SICI. However, our data show an opposite effect of MP and SES on SICI (Fig. 5). The changes in SICI are possibly related to changes in corticospinal excitability in the non-intervention M1, although absence of correlations complicates these speculations. Similarly to SICI, NMDA-mediated intracortical facilitation, ICF, did not change after motor practice and SES in line with some (Kaelin-Lang et al. 2002; Perez et al. 2004) but not with other studies (Celnik et al. 2007). In sum, it appears that SICI and ICF-related mechanisms are marginally involved in visuomotor and SES-related adaptations under the present experimental conditions. Because the methodological details of collecting the SICI, ICF, and visuomotor data were similar in the present and previous studies, the discrepancies between studies remain unclear.

#### Crossed effects on SICI

Recently, there has been a heightened interest in the role and function of the M1 ipsilateral to motor practice and sensory input (Jacobs et al. 2012; Veldman et al. 2014). In a larger perspective, these studies revealed that iM1 plasticity to short- and long-term motor and sensory interventions is a part of the adaptive network that can contribute to improved motor function as in aging (Seidler et al. 2010). In agreement with previous studies (Camus et al. 2009; Perez et al. 2007), we observed reductions in SICI in the non-intervention right-ipsilateral M1 after MP (6 %) and MP + SES (41 %) that were different from increases in SICI after SES-only intervention (−22 %; Fig. 5; interaction, *p* = 0.028). Past and current findings suggest that inhibition within the hemisphere receiving the transfer may be involved in the transfer of motor output. One mechanism could act through corticospinal excitability because the increase in corticospinal excitability correlated weakly but significantly with reductions in SICI in the non-intervention M1 (*r* = −0.37, *p* = 0.039). However, we found no relationship between changes in SICI in the non-intervention right-ipsilateral M1 and increases in motor performance in the non-intervention left hand (*r* = −0.11, *p* = 0.599), making a definitive interpretation at best speculative. In contrast with the decreased ipsilateral SICI after interventions including MP, the unique effect of SES alone on ipsilateral SICI requires special attention because we observed a non-significant 22 % (*p* = 0.144) increase instead of an expected decrease in the non-intervention M1 after SES. Based on neuroanatomical connections described earlier, the expectation is that SES-generated afferent volleys would decrease the excitability of horizontal inhibitory connections resulting in a decrease instead of an increase in SICI after SES. Our results are somewhat paradoxical because we observed a 54.2 % increase in corticospinal excitability, known to be associated with SICI, after SES. Facilitation of GABA_A_ receptors, involved in SICI (Ziemann et al. 1996), interferes with intervention-induced increases in corticospinal excitability (Butefisch et al. 2000). Considering the boundaries of the present study, we are not able to resolve this unexpected finding but one possibility is that factors other than SICI are also involved in increases in corticospinal excitability in the non-intervention M1 after SES (e.g., IHI as discussed in the next section). In contrast with SICI, ICF was not modified in both the intervention and non-intervention M1 consistent with earlier studies after motor practice (Camus et al. 2009; Uehara et al. 2013) and SES (Kaelin-Lang et al. 2002), suggesting inhibitory compared with excitatory interneurons are more sensitive to MP and SES. Taken together, ipsilateral MP and SES modified SICI differently, and the current data provide a hint that SES added to MP additionally decreases the inhibition in the ipsilateral M1 after MP. However, future studies are needed to confirm this effect and in addition examine other forms of inhibition and correlate the changes in behavior and the M1 metrics in an effort to better understand the nature of involvement of the ipsilateral M1 in motor learning.

### Interhemispheric inhibition and facilitation

During a unilateral motor task, IHI suppresses undesired activity in the ‘inactive’ hemisphere and in a bimanual task. IHI is also related to the coordination of motor activity (Hiraoka et al. 2014). Increased use of one hand can lead to interlimb transfer that concomitantly modifies IHI measured at rest and measured during a muscle contraction. For example, 1000 submaximal voluntary contractions of the right-dominant FDI produced 28 % transfer of voluntary force to the FDI of the nondominant left hand with a 31 % concomitant decrease in IHI at rest and these reductions progressively became more strongly associated with interlimb transfer (Hortobagyi et al. 2011). This association between IHI and motor performance was also shown after only a 30-min serial reaction-time task intervention (Perez et al. 2007). However, data are inconsistent because a decrease in IHI was not associated with interlimb transfer of a finger-tapping sequence (Camus et al. 2009) and IHI did not change after a complex ball rotation task (Nojima et al. 2012). In the present study, we used the iSP to quantify changes in IHI (Garvey et al. 2001; Perez et al. 2007, 2014) and observed bidirectional effects: MP increased and SES decreased IHI, respectively, by 14.2 and 7.9 % (both *p* < 0.001; Table 4; Fig. 6a) and MP + SES had no effect on IHI (1.2 % change). Similar but opposite effects were also seen for another measure of interhemispheric excitability, MEPs conditioned by a contralateral muscle contraction (contralateral facilitation, Fig. 6b). The 14.2 % increase in IHI as measured by iSP is inconsistent with some data (Perez et al. 2007) but agrees with other data (Giovannelli et al. 2009). An increase in IHI after MP would favor the interpretation that MP with one hand modifies the excitability of interhemispheric connections to preserve or even increase motor independence of the two hands. The 7.9 % decrease in IHI as measured by iSP after SES suggests that sensory input can modify the state of the non-intervention M1. Near-motor threshold SES produces afferent volleys that reach S1 and bilaterally S2 (Allison et al. 1989; Golaszewski et al. 2004; Hari et al. 1984, 1990). Consistent with previous suggestions, such inputs can modify the excitability state of the iM1 through iS2–iM1 and M1–M1 connections, giving rise to reduced IHI from stimulated to non-stimulated M1 (Shin and Sohn 2011). In view of the individual negative and positive effects of MP and SES on IHI, the unchanged IHI after MP + SES suggests the presence of a cancelation or interference effect. The neuroanatomical basis of such effects is not entirely clear because crossed effects of MP and SES are transferred through callosal fibers in the central part of the corpus callosum (Schulte and Muller-Oehring 2010) and bilateral S2 activation, respectively. Inhibitory inputs to iM1 from M1 and excitatory input from iS1 and iS2 to iM1 could sum to a net cancelation effect. We are currently examining the possibility that giving SES before instead of during MP could potentiate motor learning by priming the paths involved in IHI. Taken together, the MP and SES each can uniquely modify ipsilateral motor function in healthy young adults, with the combined effects resulting in a cancelation.

### Clinical perspective

Surprisingly, studies using SES so far have almost exclusively focussed on stroke patients, showing some promise as an adjuvant to rehabilitation of impaired motor function (Celnik et al. 2007; Conforto et al. 2007; Koesler et al. 2009; Wu et al. 2006). However, only one of these clinical trials included an age- and gender-matched healthy control group. The present study expands the clinical data by showing that SES alone or in combination with MP has specific direct and crossed effects on motor performance and neuronal excitability in healthy young adults. Although the present study did not show additive effects of SES if combined with MP in healthy adults, such an effect may be present in patients, because SES effects seem to depend on participants’ clinical status: the effects are much less (6 %) in healthy compared to stroke participants 27 % (Koesler et al. 2009). An increased understanding of the mechanisms including cortical, subcortical, and spinal involvement underlying these effects will likely contribute to an optimal protocol for the rehabilitation of patients suffering from unilateral neurological and orthopedic disorders.

### Limitations and conclusion

One limitation of the present study was that SES stimulation parameters were not systematically varied (Chipchase et al. 2011; Schabrun et al. 2012). It is possible that optimal SES parameters differ between healthy participants and patients. Second, we, as many previous studies, performed the majority of the excitability measurements at rest yet the intervention involved motor activity. Therefore, the excitability results could be different when assessed not at rest but during muscle contraction. Third, we did not perform measures of spinal excitability. Although SES does not modify F-wave characteristics (Ridding et al. 2000), we cannot completely rule out the possibility that changes in spinal excitability might have contributed to the observed effects. For example, ascending sensory and descending motor information integrate in common spinal interneurons (Nielsen 2004), possibly contributing to this involvement. Next, this study involved small groups of participants and some of the measurements revealed large variation, complicating interpretation. Finally, we did not control for environmental factors, so it is possible that changes in excitability measures are caused by experimental factors such as locus of attention or visual feedback (Poh et al. 2013).

In conclusion, MP-induced learning is most likely mediated by increased corticospinal drive at rest and during contraction. SES-induced learning is most likely the result of an upregulation of corticospinal excitability at rest possibly mediated by decreased inhibition. The physiological mechanism of transfer produced by MP remains elusive under these conditions, whereas the SES-induced transfer involves increased corticospinal excitability most likely linked to the bilateral S2 activation and its action on the M1 ipsilateral to the SES-stimulated hand. These conclusions are complicated by an absence of relevant correlations between behavioral and neuronal changes. In total, the present study showed that MP and SES each can produce motor learning and interlimb transfer but these effects are non-additive and are likely mediated by different mechanisms.

## Abbreviations

ANOVA: Analysis of variance
CLF: Contralateral facilitation
CSE: Corticospinal excitability
ECR: Extensor carpi radialis
EMG: Electromyography
ICF: Intracortical facilitation
IHI: Interhemispheric inhibition
iSP: Ipsilateral silent period
M1: Primary motor cortex
S1: Primary sensory cortex
S2: Secondary sensory cortex
MEP: Motor evoked potential
Mmax: Maximal compound action potential
MP: Motor practice
rMT: Resting motor threshold
SES: Somatosensory electrical stimulation
SICI: Short-interval intracortical inhibition
TMS: Transcranial magnetic stimulation
